# Supplementary material for: Stacking Model‐Based Classifiers for Dealing With Multiple Sets of Noisy Labels
Source: Biom J. 2025 Mar 12;67(2):e70042. doi: 10.1002/bimj.70042 (PMC11898607; doi:10.1002/bimj.70042)
Supplement: Supplementary file 1 — Supporting Information [file BIMJ-67-e70042-s001.zip › density-based-ensemble-model-main/competitors_real_data/netlab-master/netlab-master/help/rbfderiv.htm]

Netlab Reference Manual rbfderiv


# rbfderiv

## Purpose

Evaluate derivatives of RBF network outputs with respect to weights.

## Synopsis

```
g = rbfderiv(net, x)
```

## Description

`g = rbfderiv(net, x)` takes a network data structure `net`
and a matrix of input vectors `x` and returns a three-index matrix
`g` whose `i`, `j`, `k` element contains the
derivative of network output `k` with respect to weight or bias
parameter `j` for input pattern `i`. The ordering of the
weight and bias parameters is defined by `rbfunpak`. This
function also takes into account any mask in the network data structure.

## See Also

`rbf`, `rbfpak`, `rbfgrad`, `rbfbkp`

---

**Pages:**
Index


---

Copyright (c) Ian T Nabney (1996-9)
